# The Effects of Gender, Functional Condition, and ADL on Pressure Pain Threshold in Stroke Patients

**DOI:** 10.3389/fnins.2021.705516

**Published:** 2021-07-30

**Authors:** Yong-Hui Zhang, Yu-Chen Wang, Gong-Wei Hu, Xiao-Qin Ding, Xiao-Hua Shen, Hui Yang, Ji-Feng Rong, Xue-Qiang Wang

**Affiliations:** ^1^Department of Sport Rehabilitation, Shanghai University of Sport, Shanghai, China; ^2^The Center of Rehabilitation Therapy, The First Rehabilitation Hospital of Shanghai, Shanghai, China; ^3^Department of Rehabilitation Medicine, Shanghai Shangti Orthopaedic Hospital, Shanghai, China

**Keywords:** pain threshold, stroke, somatosensory, hyperalgesia, hypoalgesia, hemiplegia

## Abstract

**Background:**

Somatosensory impairments and pain are common symptoms following stroke. However, the condition of perception and pain threshold for pressure stimuli and the factors that can influence this in individuals with stroke are still unclear. This study aimed to investigate the gender differences in pressure pain threshold (PPT) and positive somatosensory signs for pressure stimuli, and explore the effects of joint pain, motor function, and activities of daily living (ADL) on pain threshold in post-stroke patients.

**Design:**

A cross-sectional study.

**Methods:**

A total of 60 participants with stroke were recruited, and their pain condition, motor functions, and ADL were evaluated by the Fugl-Meyer assessment of joint pain scale, motor function scale, and Barthel index, respectively. PPTs in eight tested points at the affected and unaffected sides were assessed.

**Results:**

Significant differences in PPTs were found between male and female patients in all measured muscles (*p* < 0.05). Positive somatosensory signs for pressure stimuli, including hypoalgesia and hyperalgesia, were frequently found at the affected side, particularly in the extremity muscles, but such signs were not significantly influenced by gender (*p* > 0.05). More equal PPTs between both sides and relatively lower PPTs at the affected side in the trunk and medial gastrocnemius muscles (*p* < 0.05) were observed in patients with less pain, better motor functions, and ADL.

**Conclusion:**

Gender differences widely exist in post-stroke survivors either at the affected or unaffected side, which are multifactorial. Sensory loss and central and/or peripheral sensitization, such as hypoalgesia and hyperalgesia for pressure stimuli, caused by a brain lesion are common signs in male and female stroke patients. Moreover, patients who are in a better condition show a more symmetrical pain sensitivity between both sides in the trunk and in female lower extremities, indicating the bidirectional improvement of somatosensory abnormalities caused by a possible neural plasticity.

## Introduction

Stroke generally affects more than 13 million people around the world yearly, and about 50% of stroke survivors have disabilities, leading to low quality of life (Collaboration, 2020; Quan et al., 2020). Somatosensory abnormality is a common symptom for post-stroke survivors, particularly ischemic strokes (Kessner et al., 2019). According to Kessner et al. (2016), the somatosensory system consists of the central and peripheral nervous systems, and it is divided into exteroception and proprioception. Several studies have reported different somatosensory abnormalities complained by stroke survivors. A study with a large sample (*n* = 207) found that 42% of post-stroke patients showed sensory impairments in light touch, temperature, or pinprick aspects (Andersen et al., 1995). Another study involving 51 stroke survivors showed that tactile and proprioceptive impairments were observed in 47 and 49% of patients’ affected side, respectively, whereas impaired tactility and proprioception were observed in 16 and 20% of patients’ unaffected side, respectively (Carey and Matyas, 2011). Considering that pain is another frequent symptom following stroke (Lundstrom et al., 2009; Plecash et al., 2019), several studies have reported patients’ sensory condition for pain stimuli with stroke through various tests, including thermal, electrical, and mechanical stimuli (Roosink et al., 2011; Soo Hoo et al., 2013; Zeilig et al., 2013; Lindgren et al., 2014). According to Maier et al. (2010), mechanical sensory abnormalities were more frequent in patients with neuropathic pain, particularly hyperalgesia for blunt pressure, and either mechanical hyperalgesia or hypoalgesia are existing in painful patients with a central nervous injury. Moreover, since PPT can be quantitatively evaluated by algometry and provide an approach to assess sensory for mechanical stimuli, it has been reported by studies regarding a different population.

For normal people, some studies pointed out that PPT could be impacted by some factors, such as gender, age, and body region (Magerl et al., 2010; Neziri et al., 2011). One study with a large sample reported that no difference in PPT was found between male and female patients (Sandrini et al., 1994). However, more studies argued that male patients normally showed a higher PPT than female patients (Chesterton et al., 2003; Magerl et al., 2010; Neziri et al., 2011). Furthermore, two studies with large sample sizes found the interaction of gender with age for PPT; of which, the increment of PPT in male patients reduced with the increase of age (Magerl et al., 2010; Neziri et al., 2011). Except for the healthy population, female patients suffering from acute or subacute neck pain, whiplash injury, or sleep loss showed a low mechanical pain tolerance than male patients (Sterling et al., 2003; Chiu et al., 2005; Walton et al., 2011b). Moreover, the correlation between a self-reported pain intensity and PPT has been reported in patients with neck pain (Walton et al., 2011b). With regard to post-stroke survivors, one study investigated the gender differences and found a higher pain sensitivity in female than male patients, but the measured points were only gathered at the shoulder region (Lin et al., 2014). Although some studies regarding PPT found that mechanical pain threshold ranged widely in stroke patients than that in the healthy control (Roosink et al., 2011, 2012; Lindgren et al., 2014), the effect of gender on the wide region remains unknown. Furthermore, two clinical studies illustrated that PPT in patients with post-stroke shoulder pain (PSSP) was lower than that without PSSP (Soo Hoo et al., 2013; Lin et al., 2014), whereas another two studies argued that no significant difference was found between stroke survivors with and without shoulder pain (Baier et al., 2014; Lindgren et al., 2014). The changing PPT in stroke patients and the factors that influence PPT remain unknown.

This study aimed to assess PPTs in eight points at the bilateral side in 60 stroke survivors and explore the gender differences. Moreover, this study aimed to research the relative somatosensory abnormalities for pressure stimuli at the affected side, the effect of gender on these abnormalities, the effects of joint pain, motor function, and ADL on PPT values, relative values at the affected side, and the symmetry between both sides.

## Materials and Methods

### Study Design

This cross-sectional study assessed PPTs at 16 symmetrical points located in the middle deltoid (MD) muscle, biceps brachii (BB) muscle, erector spinae (ES) muscles in the second and fourth lumbar vertebra (L2 and L4) levels, rectus femoris (RF) muscle, biceps femoris (BF) muscle, tibialis anterior (TA) muscle, and medial gastrocnemius (MG) muscle in stroke participants. All baseline data, including the characteristics of individual and stroke, Fugl-Meyer assessments, and Barthel Index, were assessed by one researcher and completed within 3 days prior to PPT tests.

### Participants

According to a previous study that reported the gender differences of PPT in stroke patients (Lin et al., 2014), the sample size was calculated through G^∗^Power (one tail; α, 0.05; Power, 0.80; N2/N1, 2). The results were 19 for N1 and 39 for N2, and the actual power was 0.799. Finally, this study recruited 60 stroke survivors in the First Rehabilitation Hospital of Shanghai. All patients met the inclusion and exclusion criteria presented by our researchers. The inclusion criteria were as follows: (1) aged 18–90; (2) with stroke onset between 1 month and 10 years before baseline collection; (3) with unilateral stroke caused by brain ischemia or hemorrhage; (4) with good cognitive functions that can reliably respond to pressure pain; and (5) able to maintain a prone position until the completion of PPT tests. Exclusion criteria were as follows: (1) with severe cognitive impairments that might cause late and inconsistent response to pressure stimuli; (2) with diseases that might result in somatosensory abnormalities or pain; (3) with diseases that might lead to neurological symptoms, such as multiple sclerosis, Parkinson’s disease, diabetes, or chronic low back pain; and (4) using any medicine to relieve pain. This study was approved by the Human Ethics Committee of the First Rehabilitation Hospital of Shanghai (YK-2020-01-030). All participants who were willing to attend to this study gave their written informed consent before participation.

### PPT Measurement

Pressure pain threshold was defined as the amount of pressure applied to the skin surface, allowing pressure sensation to transform into pain perception (Salom-Moreno et al., 2014; Mendigutia-Gomez et al., 2016). This study used a Force Ten Handheld Digital Force Gage (Wagner FDX-25, Greenwich, CT) to assess PPTs in all participants by the same researcher, which showed a remarkable test–retest or inter-rater reliability (intraclass correlation coefficient, 0.76–0.97) (Walton et al., 2011a). The unit of the PPT tool used in this study was kgf shorting for kgf/cm^2^, which varied from 0 to 14 kgf. We selected 16 symmetrical points widely located at different bilateral parts of the body: 12 points on the bilateral muscle bellies of MD, BB (long head), RF, BF, TA, and MG; and 4 points on the bilateral ES muscle (2 cm from the midline at the L2 and L4 levels).

All participants were introduced to the PPT test prior to the real assessments and allowed to practice their response to the pressure pain applied on the forearm muscles at the unaffected side 3–8 times until they mastered it. Otherwise, the patient was excluded from this study. In addition, we marked these 16 test points using a black pen on the skin prior to testing. All participants were asked to stay in a supine position at the beginning with arms rested in bilateral sides of the trunk to complete the PPT tests at the bilateral MD, BB, RF, and TA muscles in turn. Then, patients had to transform into the prone position with the assistance of the assessor, if necessary, to finish the PPT assessments at the L2, L4, BF, and MG muscles in turn. During the assessment, the pressure was applied by the researcher slowly and stably through the 1 cm^2^ rubber disc of the equipment on the marked points until getting the response from the participants. For all symmetrical test points, the PPT assessment was started at the unaffected side. For each point, three repetitions of the PPT tests were applied and the interval between two repetitions ranged from 20 to 40 s.

Any discomfort reported by the participants were tackled, and the participants could quit the study at any point during the whole process.

### Statistical Analyses

Data were analyzed using the IBM SPSS Statistics version 26 (IBM Corporation, Armonk, New York, United States). The baseline information, presented as frequencies and medians, was compared by the Chi-square and Mann–Whitney *U*-test between different gender groups, respectively. Given the significant results of the Shapiro–Wilk tests (*p* < 0.05), non-parametric tests were selected to detect any difference in PPTs, and *p*-values less than 0.05 were considered as a statistically significant difference.

The Mann–Whitney *U*-test was applied to compare PPTs between male and female patients. Considering that comparison between both sides is more sensitive to finding positive or negative somatosensory signs and 95% confidence intervals of relative reference data in healthy population for machinal pain threshold from 74 to 136% (Rolke et al., 2006), the PPT ratios of the affected side to the unaffected side were used to determine abnormal values. Given that the expected values of the Chi-square test were less than 5, the Fisher’s exact test was used to detect the effect of gender on somatosensory signs, including hyperalgesia, hypoalgesia, and negative sign.

Considering the significant differences between male and female patients (see “Results”), we classified the data into three groups, namely, overall, male, and female groups. We divided the data into two subgroups according to median values of the Barthel index, Fugl-Meyer assessments of joint pain, and motor function in the three groups, respectively, to determine other factors. Consequently, we obtained the below- and above-median subgroups. The Mann–Whitney *U*-test was used to find the between-subgroup differences in absolute PPT values at both sides, the PPT ratios of the affected to the unaffected side, and absolute values of differences between both sides.

## Results

### Demographics and Characteristics

The demographics and characteristics of all 60 patients (39 male and 21 female) are summarized in Table 1. Median age and BMI were 68.5 years and 23.77 kg/m^2^, respectively. All patients had a right dominant hand, and about half of them (31, 51.7%) had a right affected side. Moreover, there were gender differences in the frequencies of smoking and drinking while other baseline information were not significantly different between the male and female groups. Except for joint pain condition assessed by the Fugl-Meyer subscale, a total of 10 (16.67%) participants reported shoulder pain and 5 (8.33%) reported foot pain. None of them reported the pain influencing sleep.

**TABLE 1:** Demographics and characteristics of the participants.

	All participants (*n* = 60)	Male (*n* = 39)	Female (*n* = 21)	*p*
Age (years)	68.50 (56.25–73.00)	68.00 (55.00–71.00)	72.00 (60.00–78.00)	0.064
BMI (kg/m^2^)	23.77 (22.15–26.30)	23.88 (22.60–26.12)	23.44 (19.01–26.70)	0.466
Smoking, n (%)	26 (43.3%)	25 (64.1%)	1 (4.8%)	0.000
Drinking, n (%)	20 (33.3%)	19 (48.7%)	1 (4.8%)	0.001
Right dominant hand, n (%)	60 (100.0%)	39 (100.0%)	21 (100%)	NA
Right affected side, n (%)	31 (51.7%)	21 (53.8%)	10 (47.6%)	0.645
Stroke types, n (%)				0.182
*Hemorrhage*	21 (35.0%)	16 (41.0%)	5 (23.8%)	NA
*Ischemia*	39 (65.0%)	23 (59.0%)	16 (76.2%)	NA
Stroke onset (days)	350.00 (208.35–554.75)	353.00 (197.00–555.00)	347.00 (236.50–536.50)	0.938
FMA-MF	40.50 (25.25–76.25)	41.00 (27.00–81.00)	40.00 (24.00–66.00)	0.545
FMA-JP	40.00 (33.25–44.00)	40.00 (34.00–44.00)	41.00 (32.50–44.00)	0.994
Barthel Index	65.00 (46.25–85.00)	64.00 (45.00–85.00)	70.00 (45.00–85.00)	0.975

*BMI, body mass index; FAM, Fugl-Meyer Assessment; MF, motor function; JP, joint pain; NA, not applicable. Data are presented as median (Q1–Q3) or as number of participants (%). p-values come from the Mann–Whitney U-test for quantitative data and the Chi-square test for qualitative data.*

### PPT Values and Gender Differences

**FIGURE 1:**
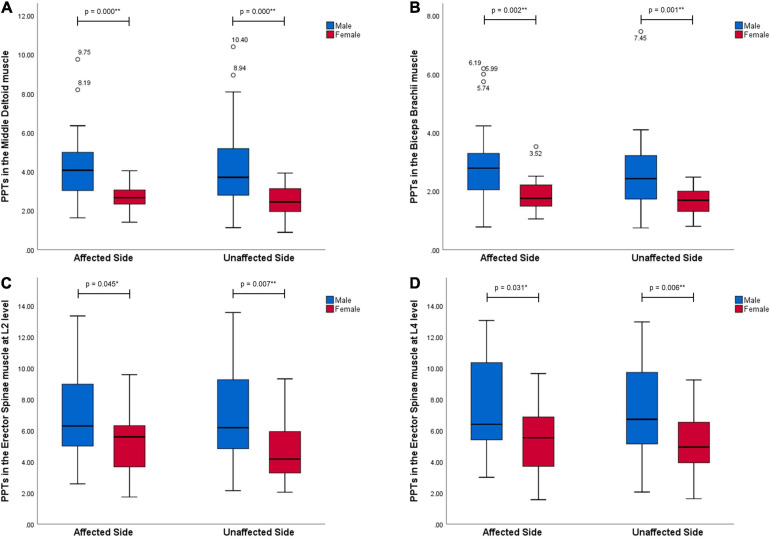
Gender differences in PPT values in the muscles in the upper extremities and trunk (kgf). All muscles, MD **(A)**, BB **(B)**, and L2 **(C)**, L4 **(D)** levels of ES, at both sides performed significantly higher pressure pain thresholds in men than women with stroke (*p* < 0.05). * Representing a statistically significant difference with *p* < 0.05; ** representing a statistically significant difference with *p* < 0.01.

**FIGURE 2:**
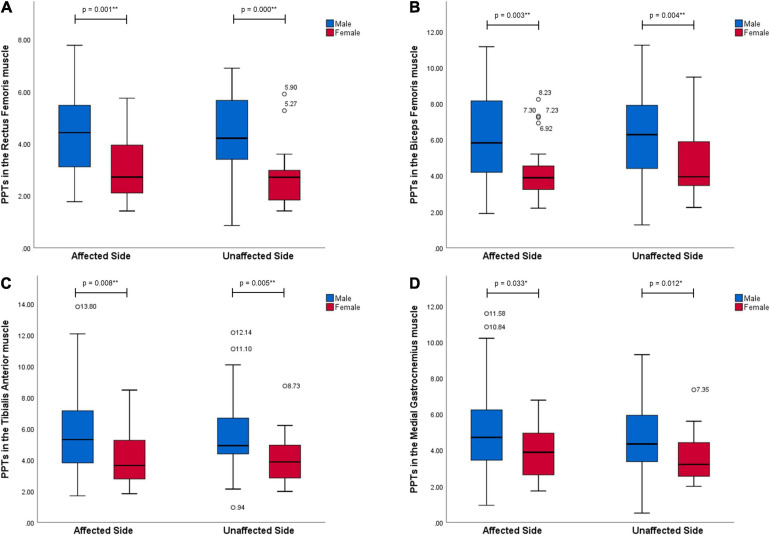
Gender differences in PPT values in the muscles in the lower extremities (kgf). All muscles, RF **(A)**, BF **(B)**, TA **(C)**, and MG **(D)**, at both sides performed significantly higher pressure pain thresholds in men than women with stroke (*p* < 0.05). * Representing a statistically significant difference with *p* < 0.05; ** representing a statistically significant difference with *p* < 0.01.

All data of PPT values are presented as median and inter quartile range (Q1–Q3) in Table 2. Figures 1, 2 show significant differences of PPTs between the male and female participants in all tested muscles with *p*-values less than 0.01 in the MD, BB, RF, BF, and TA muscles and less than 0.05 in the L2 (ES), L4 (ES), and MG muscles at both sides. Furthermore, men showed higher PPTs than women in all muscles in this study.

**TABLE 2:** Absolute pressure pain thresholds in all patients (kgf).

	All (*n* = 60)	Male (*n* = 39)	Female (*n* = 21)	*p*
**Affected side**				
MD	3.19 (2.53–4.61)	4.07 (3.01–5.02)	2.66 (2.26–3.10)	0.000
BB	2.13 (1.60–3.15)	2.78 (2.04–3.33)	1.75 (1.45–2.33)	0.002
L2	5.92 (4.31–8.67)	6.28 (4.93–9.11)	5.58 (3.54–7.03)	0.045
L4	6.09 (4.11–8.91)	6.39 (5.38–10.75)	5.52 (3.69–7.07)	0.031
RF	3.94 (2.74–5.08)	4.42 (3.09–5.47)	2.72 (2.07–3.96)	0.001
BF	4.67 (3.74–7.50)	5.82 (4.16–8.21)	3.88 (2.91–4.87)	0.003
TA	4.61 (3.36–6.53)	5.29 (3.79–7.40)	3.63 (2.68–5.30)	0.008
MG	4.25 (3.33–5.88)	4.71 (3.41–6.31)	3.88 (2.54–5.01)	0.033
**Unaffected side**				
MD	3.12 (2.34–4.45)	3.70 (2.76–5.25)	2.44 (1.92–3.15)	0.000
BB	2.00 (1.58–2.83)	2.42 (1.71–3.21)	1.69 (1.27–2.14)	0.001
L2	5.79 (3.77–8.70)	6.17 (4.80–9.31)	4.16 (3.17–6.24)	0.007
L4	5.77 (4.11–8.25)	6.70 (5.12–10.12)	4.93 (3.62–6.71)	0.006
RF	3.52 (2.61–5.26)	4.21 (3.39–5.89)	2.71 (1.83–2.99)	0.000
BF	5.53 (3.93–7.11)	6.28 (4.39–8.16)	3.93 (3.29–5.91)	0.004
TA	4.75 (3.43–6.00)	4.91 (4.36–6.77)	3.86 (2.62–5.05)	0.005
MG	4.02 (2.84–5.49)	4.34 (3.28–5.99)	3.21 (2.49–4.43)	0.012

*MD, middle deltoid muscle; BB, biceps brachii muscle; L2 and L4, erector spinae muscle at L2 and L4 levels; RF, rectus femoris muscle; BF, biceps femoris muscle; TA, tibialis anterior muscle; MG, medial gastrocnemius muscle.*

*Data are presented as median (Q1–Q3). p-values come from the Mann–Whitney U-test.*

### Positive Somatosensory Signs

According to the threshold ratios, relative abnormalities at the affected side and its percentage are presented in Table 3. Although hypoalgesia and hyperalgesia were both observed, hypoalgesia appeared more frequently in the BB, ES, RF, TA, and MG muscles than hyperalgesia. Furthermore, the MD, BB, RF, BF, and MG muscles showed a higher risk of pain threshold abnormalities (over 15%) than the other muscles. With regard to gender, female participants showed higher risks of positive sign than male participants in the MD, BB, L2 (ES), RF, BF, and MG muscles but lower risks in the L4 (ES) and TA muscles. In detail, female participants showed a higher percentage of hypoalgesia in the MD, BB, L2 (ES), RF, BF, and MG muscles and hyperalgesia in the L4 (ES), BF, and MG muscles than male participants. However, no gender difference was detected by the Fisher’s exact test (*p* > 0.05).

**TABLE 3:** Somatosensory signs for pressure stimuli (n,%).

	Positive signs			Negative sign	*p*-values
	Hypoalgesia	Hyperalgesia	All		
**Male (*n* = 39)**					
MD	3 (7.69%)	6 (15.38%)	9 (23.08%)	30 (76.92%)	0.263
BB	7 (17.95%)	3 (7.69%)	10 (25.64%)	29 (74.36%)	0.650
L2	3 (7.69%)	0 (0)	3 (7.69%)	36 (92.31%)	1.000
L4	3 (7.69%)	1 (2.56%)	4 (10.26%)	35 (89.74%)	1.000
RF	3 (7.69%)	2 (5.13%)	5 (12.82%)	34 (87.18%)	0.221
BF	3 (7.69%)	1 (2.56%)	4 (10.26%)	35 (89.74%)	0.690
TA	5 (12.82%)	0 (0)	5 (12.82%)	34 (87.18%)	1.000
MG	4 (10.26%)	1 (2.56%)	5 (12.82%)	34 (87.18%)	0.411
**Female (*n* = 21)**					
MD	4 (19.05%)	1 (4.76%)	5 (23.81%)	16 (76.19%)	NA
BB	6 (28.57%)	1 (4.76%)	7 (33.33%)	14 (66.67%)	NA
L2	2 (9.52%)	0 (0)	2 (9.52%)	19 (90.48%)	NA
L4	1 (4.76%)	1 (4.76%)	2 (9.52%)	19 (90.48%)	NA
RF	5 (23.81%)	0 (0)	5 (23.81%)	16 (76.19%)	NA
BF	2 (9.52%)	4 (19.05%)	6 (28.57%)	15 (71.43%)	NA
TA	2 (9.52%)	0 (0)	2 (9.52%)	19 (90.48%)	NA
MG	3 (14.29%)	2 (9.52%)	5 (23.81%)	16 (76.19%)	NA
**All participants (*n* = 60)**					
MD	7 (11.67%)	7 (11.67%)	14 (23.33%)	46 (76.67%)	NA
BB	13 (21.67%)	4 (6.67%)	17 (28.33%)	43 (71.67%)	NA
L2	5 (8.33%)	0 (0)	5 (8.33%)	55 (91.67%)	NA
L4	4 (6.67%)	2 (3.33%)	6 (10.00%)	54 (90.00%)	NA
RF	8 (13.33%)	2 (3.33%)	10 (16.67%)	50 (83.33%)	NA
BF	5 (8.33%)	5 (8.33%)	10 (16.67%)	50 (83.33%)	NA
TA	7 (11.67%)	0 (0)	7 (11.67%)	53 (88.33%)	NA
MG	7 (11.67%)	3 (5.00%)	10 (16.67%)	50 (83.33%)	NA

*MD, middle deltoid muscle; BB, biceps brachii muscle; L2 and L4, erector spinae muscle at L2 and L4 levels; RF, rectus femoris muscle; BF, biceps femoris muscle; TA, tibialis anterior muscle; MG, medial gastrocnemius muscle; NA, not applicable.*

*Data are presented as number of subjects (% of male, female or all participants). Positive signs were determined by the ratios of PPTs (affected/unaffected). Hypoalgesia: ratio > 136%; Hyperalgesia: ratio < 74%; p-values come from the Fisher’s exact test used to detect the influence of gender on the frequencies of hyperalgesia, hypoalgesia, and negative sign but no significant differences were found.*

### Other Factors for PPT

All results from independent comparisons between the below- and above-median subgroups based on clinically functional assessments are shown in the Supplementary File (Supplementary Table 1 for the overall group, Supplementary Table 2 for the male group, and Supplementary Table 3 for the female group), and significant differences (*p* < 0.05) are presented in Table 4. According to the Fugl-Meyer assessment of joint pain, the above-median subgroup showed significantly decreased differences between both sides in the L2 and L4 levels of ES muscles in the overall group. In the Fugl-Meyer assessment of motor function, the above-median subgroup showed a lower difference between the two sides in the MG muscle and lower PPT ratios in the L4 and MG muscles compared to the below-median subgroup. However, the above-median subgroup in the male group showed a higher PPT in L2 at the unaffected side and a lower PPT ratio in the L4 muscle than the below-median subgroup, whereas that in the female group showed lower ratios in the MG muscle and less difference between sides in the L4 and MG muscles. As shown in the Barthel Index, the above-median subgroup had a lower ratio and absolute difference than the below-median subgroup in the MG muscle.

**TABLE 4:** All significant between-group differences based on the medians of the assessment scales (kgf or%).

	Overall group (*n* = 60)			Male group (*n* = 39)			Female group (*n* = 21)		
		
	Below-median	Above-median	p1	Below-median	Above-median	p2	Below-median	Above-median	p3
***FMA-JP***	***28 (19, 67.86%)^#^***	***32 (20, 62.5%)^#^***		***n* = *19***	***n* = *20***		***n* = *10***	***n* = *11***	
L2 (diff)	0.77 (0.42–1.41)	0.51 (0.27–0.90)	0.015*	0.74 (0.49–1.19)	0.67 (0.31–1.17)	NS	0.80 (0.33–1.87)	0.28 (0.15–0.61)	0.020*
L4 (diff)	0.82 (0.57–1.27)	0.49 (0.22–0.71)	0.006**	0.84 (0.62–1.29)	0.43 (0.15–0.70)	0.013*	0.65 (0.49–1.28)	0.54 (0.34–0.79)	NS
***FMA-MF***	***30 (19, 63.33%)^#^***	***30 (20, 66.67%)^#^***		***n* = *19***	***n* = *20***		***n* = *10***	***n* = *11***	
L2 (UA)	5.35 (3.58–6.59)	6.16 (4.39–9.87)	NS	5.68 (4.18–8.51)	8.07 (5.28–10.77)	0.033*	4.11 (3.22–6.59)	4.32 (3.08–5.92)	NS
L4 (diff)	0.74 (0.33–1.35)	0.55 (0.23–0.79)	NS	0.70 (0.26–1.69)	0.64 (0.26–0.92)	NS	0.93 (0.56–1.28)	0.51 (0.14–0.65)	0.024*
L4 (%)	112 (100–122)	102 (92–108)	0.030*	109 (100–127)	100 (89–106)	0.016*	116 (90–122)	110 (100–118)	NS
MG (diff)	0.83 (0.43–1.18)	0.50 (0.22–0.91)	0.024*	0.72 (0.37–1.15)	0.61 (0.24–0.97)	NS	1.07 (0.67–1.21)	0.33 (0.11–0.89)	0.002**
MG (%)	122 (97–132)	99 (92–117)	0.027*	113 (99–127)	109 (94–122)	NS	129 (92–154)	93 (88–102)	0.020*
***Barthel***	***27 (20, 74.07%)^#^***	***33 (19, 57.58%)^#^***		***n* = *19***	***n* = *20***		***n* = *10***	***n* = *11***	
MG (A)	4.71 (3.52–6.08)	3.97 (2.76–5.57)	NS	4.51 (3.39–6.08)	5.20 (3.51–7.39)	NS	4.19 (3.69–5.60)	2.63 (2.20–4.46)	0.043*
MG (diff)	0.98 (0.58–1.19)	0.45 (0.22–0.89)	0.008**	0.72 (0.37–1.47)	0.51 (0.24–0.92)	NS	1.02 (0.54–1.18)	0.55 (0.11–0.92)	0.043*
MG (%)	124 (105–131)	96 (91–116)	0.002**	119 (105–129)	100 (93–118)	0.044*	126 (92–138)	96 (88–116)	NS

*FAM, Fugl-Meyer Assessment; JP, joint pain; MF, motor function; L2 and L4, erector spinae muscle at L2 and L4 levels; MG, medial gastrocnemius muscle; diff, absolute values of (affected—unaffected) (kgf);%, ratio of affected/unaffected; UA, PPT values at the unaffected side; A, PPT values at the affected side; p1, p2, and p3, p-values from the Mann-Whitney U-tests in overall group, male and female groups, respectively, NS, not significant (p > 0.05).*

Data are presented as median (Q1–Q3). *Representing a statistically significant difference with p < 0.05; **representing a statistically significant difference with p < 0.01; # number of all participants in subgroup (numbers of male in subgroup, male percentage of all participants in subgroup).

## Discussion

This study investigated the gender differences in mechanical pain sensitivity and somatosensory abnormalities for pressure stimuli and other factors for pain threshold in stroke patients. We found that the female participants showed a higher mechanical pain sensitivity than male participants at the affected and unaffected sides in the large muscles all over the body. The affected side showed somatosensory abnormalities that were related to hypoalgesia and hyperalgesia, and the positive sign was not affected by gender. Furthermore, stroke patients with less pain, greater motor functions, and ADL had different changes of absolute pain threshold in different muscles, reduced relative pain threshold, and more symmetrical mechanical pain thresholds between the two sides in the trunk and medial gastrocnemius muscles.

### Effect of Gender

Female patients showed a significantly higher mechanical pain sensitivity than male patients in all the tested muscles at both sides. This gender difference has been reported in the healthy population (Chesterton et al., 2003), people suffering from poor sleep and depression (Chiu et al., 2005), and various patients suffering from pain (Sterling et al., 2003; Walton et al., 2011b). This study provided a new evidence for the high pain sensitivity among female patients with stroke and suggested that gender differences generally existed in large muscles all over the body. The explanation for the gender difference could be multifactorial. Firstly, although some studies pointed out that similar endogenous pain regulatory mechanisms were found in the male and female populations (Tousignant-Laflamme et al., 2008; Weissman-Fogel et al., 2008), several studies argued that endogenous pain inhibitory systems were more effective in males than in females, thereby a higher sensitivity for pain in females (Granot et al., 2008; Bulls et al., 2015). Furthermore, the gender differences in the central processing of nociception may be another reason for a lower PPT in the female population, because some studies have demonstrated different brain-evoked potentials during pain stimuli between male and female subjects (Chapman et al., 1999; Lundstrom et al., 2005). Similarly, studies focusing on the periaqueductal gray that involves pain regulation stated that male people might show a higher recruitment of periaqueductal gray and amygdala inhibition circuitry with an increasing pain stimuli than female (Loyd and Murphy, 2009; Linnman et al., 2012). Except for a biological reason, psychological and sociological factors, such as depression status and family and social roles, may also lead to gender differences in pain threshold (Racine et al., 2012).

With regard to pressure perception in this study, hypoalgesia and hyperalgesia existed in widespread muscles in post-stroke patients. In addition, the prevalence of hyperalgesia was normally lower than hypoalgesia, particularly in the BB, RF, TA, and MG muscles, which was similar to a study suggesting that the distal extremities might suffer from a more serious post-stroke sensory impairment than the proximal shoulder girdle (Lin et al., 2014). These positive somatosensory signs have also been demonstrated in some studies finding positive hyperalgesia and hypoalgesia for pressure stimuli and wide ranges in mechanical pain threshold in post-stroke patients with or without shoulder pain (Roosink et al., 2011; Lindgren et al., 2014). Similarly, some studies also stated that disorders of pain sensation that is likely caused by a damaged spinothalamic tract are also common in stroke patients without pain (Finnerup et al., 2003; Ducreux et al., 2006). The positive somatosensory abnormalities could be associated with brain lesions of the somatosensory cortex or a damaged nervous system that processes pressure and pain stimuli. According to a review study by Klit et al. (2009), the pathophysiological mechanisms seem to be different due to the various locations of the brain lesion in stroke patients, and the association between lesions and clinical symptoms is still unsure. Consequently, the possible interpretation for the nervous system mechanisms of stroke should consider the clinical symptoms. On the one hand, somatosensory loss and hypoalgesia for pressure perception were mostly found in post-stroke patients, which could be caused by deafferentation. On the other hand, since the central and/or peripheral sensitization might also develop in stroke patients without neuropathic pain (Roosink et al., 2012), pain sensitization as one of the reasons leading to hyperalgesia cannot be ruled out in this study. Furthermore, the risks of somatosensory abnormalities seemed to be independent of gender. Although female participants showed a slightly higher prevalence of positive signs than male participants in six out of eight muscles, the differences were not significant. This finding is supported by the evidence that gender as a factor cannot predict the occurrence of central pain after stroke (Andersen et al., 1995) and the gender difference is not existing in peripheral sensitization after a high pain stimuli (Ge et al., 2004, 2006).

### Effects of Functions and ADL

No difference in mechanical pain threshold was found between less and more pain subgroups based on the joint pain subscale of Fugl-Meyer among the female, male, and overall groups. Although findings from two studies supported that mechanical pain thresholds were approximately equal between painful and pain-free stroke patients (Baier et al., 2014; Lindgren et al., 2014), more studies argued that an increased pain sensitivity was associated with persistent PSSP, and pressure pain tolerance was negatively related to pain intensity of the shoulder (Roosink et al., 2011; Soo Hoo et al., 2013; Lin et al., 2014). The distinct finding of this study could be explained by the different properties and locations of pain in the participants because the Fugl-Meyer assessment subscale evaluates the pain during movement at most joints. Moreover, previous studies mostly focused on the deltoid muscles, whereas this study measured more muscles in different body parts. This finding may indicate that the hyperalgesia for pressure stimuli based on central sensitization is independent of joint pain states during movement in stroke patients. The central sensitization after stroke could be caused by the increased neuronal excitability, loss of inhibition, and facilitation following brain lesions (Klit et al., 2009), whereas joint pain during movement is likely associated with spasticity and muscle disuse or overuse (Fernandez-de-las-Penas et al., 2009). Furthermore, we detected decreased difference values between two sides among patients in better pain conditions, indicating that a better condition of pain was associated with a more symmetrical mechanical pain sensitivity between the two sides in erector spine muscles. Similarly, previous studies found that the extent of increment in PPT at the affected side with respect to the unaffected side was reduced in non-PSSP patients compared to PSSP patients, which was consistent with the results in this study despite the different muscles tested (Roosink et al., 2011, 2012). From this perspective, improvement of joint pain condition could increase the symmetry of pressure pain sensitivities at two sides, which indicated the recovery of somatosensory abnormities after stroke.

Based on the motor function and ADL, an increase of mechanical pain tolerance in male trunk muscles but a decrease of pain tolerance in female calf muscles was found in patients with a better condition. The trends of absolute pain threshold in the different muscles were conflicting. However, the trends of relative pain sensitivity were the same in the trunk and calf muscles, which were decreased relative mechanical pain sensitivity at the affected side in patients with a better motor function or higher ADL. At the same time, the difference value between the PPTs at the two sides in these muscles was significantly reduced in these patients. These findings suggested that stroke patients with higher abilities of motor and daily activities appeared with more symmetrical pain thresholds between the two body sides.

From the perspectives of joint pain, motor function, and ADL, patients in better conditions showed more symmetrical PPTs between the two sides than those in worse conditions. In the presence of hypoalgesia and hyperalgesia, this finding suggested that the recovery of joint pain, functions, and ADL is associated with the bidirectional improvement of pain sensitivity in stroke patients. Firstly, for the recovery of hyperalgesia, the good motor functions and ADL, indicating more physical activities, could reduce pain sensitivity based on the finding of exercise-induced hypoalgesia (Chiu and Wright, 1996; Fernandez-de-Las-Penas et al., 2008; Soon et al., 2010). Reciprocally, some studies suggested that a higher pain threshold is related to high-efficient diffuse noxious inhibitory control and endogenous pain inhibitory processes, and the higher efficiency of pain inhibitory systems is a predictor of less clinical pain and higher mobility (Edwards et al., 2003a, b). In this study, this higher mobility means that stroke patients have a better motor function and higher ADL score. Secondly, from the perspective of hypoalgesia recovery, studies regarding sensory loss in stroke patients reported that a weak-to-moderate correlation between tactile sensation, proprioception and motor function, and ADL, with an increased sensory related to a better physical function and ADL (de Haart et al., 2004; Marigold et al., 2004; Lin, 2005; Tyson et al., 2008). The possible reason is that sensory loss is a direct or indirect negative factor for the recovery of motor and ADL outcomes (Tyson et al., 2008; Stinear, 2010). Overall, the bidirectional improvement of PPTs related to less pain, better motor function, and ADL, which could be a result of neural plasticity, indicating that the recovery of somatosensory abnormities, including hyperalgesia and hypoalgesia. With regard to the body region, muscles in the trunk and lower extremities were more sensitive to the improvement of motor functions compared with the other muscles, which could be interpreted by their important roles in the kinematic chain, balance, and mobility. Consistent with the higher prevalence of positive somatosensory signs in the MG muscles in female than that in male participants and similar prevalence in the trunk muscles between male and female participants, the PPTs of the MG muscles in the female group and trunk muscles in both gender groups were sensitive to change following motor functions in stroke patients.

### Limitations

This study has some limitations. First, the sample size was small, particularly the number of the female subgroup. Second, the participants recruited were mostly in good functional and psychological conditions to ensure the reliability of the response to pressure pain. Therefore, the clinical assessment results of these participants were concentrated in a high level, which might affect the results of between-group comparison. For example, non-parametric analyses might miss some important values. In addition, although we compared PPTs in all muscles between ischemic and hemorrhagic stroke patients, no difference was detected. Considering that the symptoms of somatosensory deficits seemed to be different based on the affected region of brain lesion (Meyer et al., 2016), a detailed investigation on the subtypes of ischemic stroke might provide more information and explanation about the results of PPTs. Finally, although we evaluated factors for PPT in stroke patients from different perspectives, including gender, joint pain conditions, motor functions, and ADL, other factors might still affect PPT, such as instrumental ADL, depression status, muscle strength, and physical activities before stroke.

### Clinical Implications and Future Research

Mechanical pain sensitivity in post-stroke survivors is associated with gender, motor functions, ADL, and pain conditions. The somatosensory deficits are individualized and multifactorial, and neglect of these abnormalities in the management plan may inhibit the recovery of mobility and pain. Therefore, physiotherapists must pay more attention to and take some specific therapy for specific somatosensory impairments, including pressure perception loss, hypoalgesia, and hyperalgesia for pressure stimuli, in post-stroke patients to prevent worse pain and motor conditions. Additionally, motor therapy combined with sensory training may positively impact somatosensory impairments through neural plasticity, thereby improving symptoms of hypoalgesia and hyperalgesia. Further studies with a larger sample size, stricter recruitment criteria, and quantitative measurements are required to explore the changes of various pain thresholds and their correlation with brain activities in patients with different types of stroke, thereby providing evidence for the clinical application of this management plan.

In conclusion, female stroke patients show a higher mechanical pain sensitivity than male stroke patients in large muscles all over the body, which may be caused by biological, psychological, or/and sociological factors, and the abnormalities for pressure perception in stroke patients are widespread and multifactorial but not significantly affected by gender. Hypoalgesia and hyperalgesia for pressure stimuli are common symptoms found at the affected side of patients, particularly in the upper and lower extremity muscles, which can be due to a brain lesion or followed central and/or peripheral sensitization or both. Furthermore, in this study, patients with less pain, better motor functions, and ADL showed a relatively decreased pain tolerance at the affected side and more symmetrical pain sensitivity between the two sides in the female medial gastrocnemius muscles and the female and male trunk muscles, which might indicate that good pain condition and functions were associated with a bidirectional improvement of pain threshold abnormalities because of neural plasticity, meaning the simultaneous recovery of hypoalgesia and hyperalgesia.

## Data Availability Statement

The original contributions presented in the study are included in the article/Supplementary Material, further inquiries can be directed to the corresponding author/s.

## Ethics Statement

The studies involving human participants were reviewed and approved by the Human Ethics Committee of The First Rehabilitation Hospital of Shanghai (YK-2020-01-030). The patients/participants provided their written informed consent to participate in this study.

## Author Contributions

J-FR and X-QW: conceptualization. Y-HZ, Y-CW, G-WH, X-QD, X-HS, and HY: investigation. Y-HZ, G-WH, X-QD, X-HS, and HY: data curation and analysis. Y-CW and Y-HZ: validation and writing—original draft preparation. Y-HZ and X-QW: writing—review and editing. X-QW: supervision. All authors have read and agreed to the published version of the manuscript.

## Conflict of Interest

The authors declare that the research was conducted in the absence of any commercial or financial relationships that could be construed as a potential conflict of interest.

## Publisher’s Note

All claims expressed in this article are solely those of the authors and do not necessarily represent those of their affiliated organizations, or those of the publisher, the editors and the reviewers. Any product that may be evaluated in this article, or claim that may be made by its manufacturer, is not guaranteed or endorsed by the publisher.
