# Supplementary material for: The Effects of Gender, Functional Condition, and ADL on Pressure Pain Threshold in Stroke Patients
Source: Front Neurosci. 2021 Jul 30;15:705516. doi: 10.3389/fnins.2021.705516 (PMC8366776; doi:10.3389/fnins.2021.705516)
Supplement: Supplementary file 1 [file Data_Sheet_1.docx]

Supplementary Material

**Table S1.** All results from between-subgroup comparison based on medians of assessment scales in all participants

|  | FMA-JP | | | FMA-MF | | | Barthel Index | |
| --- | --- | --- | --- | --- | --- | --- | --- | --- |
|  | z | p | z | | p | z | | p |
| MD (UA) | -0.059 | 0.953 | -1.434 | | 0.152 | -0.104 | | 0.917 |
| MD (A) | -0.141 | 0.888 | -0.658 | | 0.511 | -0.193 | | 0.847 |
| BB (UA) | -0.682 | 0.495 | -0.643 | | 0.520 | 0.000 | | 1.000 |
| BB (A) | -0.274 | 0.784 | -0.577 | | 0.564 | -0.067 | | 0.947 |
| L2 (UA) | -0.630 | 0.529 | -1.907 | | 0.056 | -0.319 | | 0.749 |
| L2 (A) | -0.585 | 0.558 | -1.146 | | 0.252 | -0.305 | | 0.761 |
| L4 (UA) | -0.459 | 0.646 | -1.641 | | 0.101 | -0.037 | | 0.970 |
| L4 (A) | -0.333 | 0.739 | -0.909 | | 0.363 | -0.238 | | 0.812 |
| RF (UA) | -0.370 | 0.711 | -0.828 | | 0.408 | -0.602 | | 0.547 |
| RF (A) | -0.267 | 0.790 | -0.237 | | 0.813 | -0.944 | | 0.345 |
| BF (UA) | -0.563 | 0.573 | -1.146 | | 0.252 | -0.334 | | 0.738 |
| BF (A) | -0.044 | 0.965 | -0.843 | | 0.399 | -0.632 | | 0.528 |
| TA (UA) | -0.044 | 0.965 | -0.902 | | 0.367 | -0.490 | | 0.624 |
| TA (A) | -0.230 | 0.818 | -0.259 | | 0.796 | -1.152 | | 0.249 |
| MG (UA) | -0.341 | 0.733 | -0.769 | | 0.442 | -0.067 | | 0.947 |
| MG (A) | -0.296 | 0.767 | -0.207 | | 0.836 | -1.330 | | 0.184 |
| MD (diff) | -0.378 | 0.706 | -0.355 | | 0.723 | -1.018 | | 0.309 |
| MD (%) | -0.919 | 0.358 | -1.538 | | 0.124 | -1.300 | | 0.194 |
| BB (diff) | -1.082 | 0.279 | -0.311 | | 0.756 | -0.275 | | 0.783 |
| BB (%) | -1.748 | 0.080 | -0.089 | | 0.929 | -0.305 | | 0.761 |
| L2 (diff) | -2.430 | 0.015* | -0.628 | | 0.530 | -0.669 | | 0.504 |
| L2 (%) | -0.030 | 0.976 | -1.582 | | 0.114 | -0.260 | | 0.795 |
| L4 (diff) | -2.734 | 0.006** | -1.419 | | 0.156 | -1.917 | | 0.055 |
| L4 (%) | -1.126 | 0.260 | -2.173 | | 0.030* | -1.389 | | 0.165 |
| RF (diff) | -0.326 | 0.744 | -0.229 | | 0.819 | -1.390 | | 0.165 |
| RF (%) | -0.267 | 0.790 | -1.523 | | 0.128 | -1.122 | | 0.262 |
| BF (diff) | -0.822 | 0.411 | -0.067 | | 0.947 | -0.334 | | 0.738 |
| BF (%) | -0.519 | 0.604 | -0.059 | | 0.953 | -1.300 | | 0.194 |
| TA (diff) | -1.074 | 0.283 | -0.835 | | 0.404 | -0.914 | | 0.361 |
| TA (%) | -0.282 | 0.778 | -0.887 | | 0.375 | -1.196 | | 0.232 |
| MG (diff) | -0.667 | 0.505 | -2.262 | | 0.024* | -2.660 | | 0.008** |
| MG (%) | -0.015 | 0.988 | -2.218 | | 0.027* | -3.068 | | 0.002** |

Abbreviations: FAM, Fugl-Meyer Assessment; JP, joint pain; MF, motor function; MD, middle deltoid muscle; BB, biceps brachii muscle; L2 and L4, erector spinae muscle at L2 and L4 levels; RF, rectus femoris muscle; BF, biceps femoris muscle; TA, tibialis anterior muscle; MG, medial gastrocnemius muscle; A, affected side; UA, unaffected side; diff, absolute values of (affected - unaffected); %, ratio of affected/unaffected; z and p, z and p values from the Mann-Whitney U tests.

NOTE: * representing a statistically significant difference with p < 0.05; ** representing a statistically significant difference with p < 0.01.

**Table S2.** All results from between-subgroup comparison based on medians of assessment scales in male participants

|  | FMA-JP | | | FMA-MF | | | Barthel Index | |
| --- | --- | --- | --- | --- | --- | --- | --- | --- |
|  | z | p | z | | p | z | | p |
| MD (UA) | -0.211 | 0.835 | -1.110 | | 0.270 | -1.658 | | 0.101 |
| MD (A) | -0.014 | 0.989 | -0.913 | | 0.365 | -1.419 | | 0.158 |
| BB (UA) | -0.169 | 0.879 | -0.478 | | 0.647 | -1.040 | | 0.309 |
| BB (A) | -0.379 | 0.708 | -0.337 | | 0.749 | -0.899 | | 0.380 |
| L2 (UA) | -0.590 | 0.569 | -2.135 | | 0.033* | -1.096 | | 0.283 |
| L2 (A) | -0.885 | 0.380 | -1.391 | | 0.166 | -0.983 | | 0.336 |
| L4 (UA) | -0.590 | 0.569 | -1.883 | | 0.061 | -1.292 | | 0.204 |
| L4 (A) | -0.351 | 0.728 | -1.250 | | 0.214 | -1.236 | | 0.224 |
| RF (UA) | 0.000 | 1.000 | -1.012 | | 0.322 | -0.927 | | 0.365 |
| RF (A) | -0.028 | 0.989 | -0.618 | | 0.550 | -0.759 | | 0.461 |
| BF (UA) | -0.618 | 0.550 | -1.489 | | 0.141 | -1.517 | | 0.134 |
| BF (A) | -0.590 | 0.569 | -1.292 | | 0.204 | -0.927 | | 0.365 |
| TA (UA) | -0.014 | 0.989 | -0.998 | | 0.322 | -0.506 | | 0.627 |
| TA (A) | -0.042 | 0.967 | -0.745 | | 0.461 | -0.421 | | 0.687 |
| MG (UA) | -0.590 | 0.569 | -1.096 | | 0.283 | -1.321 | | 0.194 |
| MG (A) | -0.759 | 0.461 | -0.899 | | 0.380 | -0.646 | | 0.531 |
| MD (diff) | -0.632 | 0.531 | -0.365 | | 0.728 | -0.759 | | 0.461 |
| MD (%) | -0.506 | 0.627 | -0.702 | | 0.496 | -1.826 | | 0.070 |
| BB (diff) | -0.984 | 0.336 | -0.927 | | 0.365 | -0.871 | | 0.396 |
| BB (%) | -1.208 | 0.235 | -0.421 | | 0.687 | -0.197 | | 0.857 |
| L2 (diff) | -0.759 | 0.461 | -0.028 | | 0.989 | -0.337 | | 0.749 |
| L2 (%) | -0.815 | 0.428 | -1.714 | | 0.089 | -0.534 | | 0.607 |
| L4 (diff) | -2.473 | 0.013* | -0.253 | | 0.813 | -0.421 | | 0.687 |
| L4 (%) | -0.843 | 0.411 | -2.388 | | 0.016* | -0.731 | | 0.478 |
| RF (diff) | -0.660 | 0.513 | -0.506 | | 0.627 | -1.236 | | 0.224 |
| RF (%) | -0.112 | 0.923 | -1.124 | | 0.270 | -0.702 | | 0.496 |
| BF (diff) | -1.264 | 0.214 | -0.056 | | 0.967 | -0.815 | | 0.428 |
| BF (%) | -0.337 | 0.749 | -0.197 | | 0.857 | -0.927 | | 0.365 |
| TA (diff) | -0.084 | 0.945 | -0.393 | | 0.708 | -0.745 | | 0.461 |
| TA (%) | -0.365 | 0.728 | -0.421 | | 0.687 | -0.140 | | 0.901 |
| MG (diff) | -0.197 | 0.857 | -0.421 | | 0.687 | -0.927 | | 0.365 |
| MG (%) | -0.253 | 0.813 | -0.562 | | 0.588 | -2.023 | | 0.044* |

Abbreviations: FAM, Fugl-Meyer Assessment; JP, joint pain; MF, motor function; MD, middle deltoid muscle; BB, biceps brachii muscle; L2 and L4, erector spinae muscle at L2 and L4 levels; RF, rectus femoris muscle; BF, biceps femoris muscle; TA, tibialis anterior muscle; MG, medial gastrocnemius muscle; A, affected side; UA, unaffected side; diff, absolute values of (affected - unaffected); %, ratio of affected/unaffected; z and p, z and p values from the Mann-Whitney U tests.

NOTE: * representing a statistically significant difference with p < 0.05; ** representing a statistically significant difference with p < 0.01.

**Table S3.** All results from between-subgroup comparison based on medians of assessment scales in female participants

|  | FMA-JP | | | FMA-MF | | | Barthel Index | |
| --- | --- | --- | --- | --- | --- | --- | --- | --- |
|  | z | p | z | | p | z | | p |
| MD (UA) | -1.197 | 0.251 | -0.352 | | 0.756 | -0.423 | | 0.705 |
| MD (A) | -1.268 | 0.223 | -0.493 | | 0.654 | -0.563 | | 0.605 |
| BB (UA) | -1.549 | 0.132 | -0.141 | | 0.918 | -0.493 | | 0.654 |
| BB (A) | -0.986 | 0.349 | -0.141 | | 0.918 | -0.634 | | 0.557 |
| L2 (UA) | -0.211 | 0.863 | -0.211 | | 0.863 | -0.141 | | 0.918 |
| L2 (A) | -0.634 | 0.557 | -0.211 | | 0.863 | -0.070 | | 0.973 |
| L4 (UA) | -0.141 | 0.918 | -0.211 | | 0.863 | -0.141 | | 0.918 |
| L4 (A) | -0.211 | 0.863 | -0.070 | | 0.973 | 0.000 | | 1.000 |
| RF (UA) | -0.211 | 0.863 | -0.634 | | 0.557 | -0.423 | | 0.705 |
| RF (A) | -0.423 | 0.705 | -0.634 | | 0.557 | -0.775 | | 0.468 |
| BF (UA) | -0.352 | 0.756 | -0.141 | | 0.918 | -0.423 | | 0.705 |
| BF (A) | -0.915 | 0.387 | -0.915 | | 0.387 | -0.845 | | 0.426 |
| TA (UA) | -0.282 | 0.809 | -0.035 | | 0.973 | -0.528 | | 0.605 |
| TA (A) | -0.493 | 0.654 | -0.845 | | 0.426 | -0.915 | | 0.387 |
| MG (UA) | -0.528 | 0.605 | -0.070 | | 0.973 | -1.127 | | 0.282 |
| MG (A) | -0.915 | 0.387 | -1.690 | | 0.099 | -2.042 | | 0.043* |
| MD (diff) | -0.070 | 0.973 | -0.986 | | 0.349 | -0.775 | | 0.468 |
| MD (%) | -0.704 | 0.512 | -1.268 | | 0.223 | -0.352 | | 0.756 |
| BB (diff) | -0.528 | 0.605 | -0.845 | | 0.426 | -0.704 | | 0.512 |
| BB (%) | -0.634 | 0.557 | -0.211 | | 0.863 | -0.775 | | 0.468 |
| L2 (diff) | -2.289 | 0.020* | -0.387 | | 0.705 | -0.247 | | 0.809 |
| L2 (%) | -1.479 | 0.152 | -0.352 | | 0.756 | -1.408 | | 0.173 |
| L4 (diff) | -0.845 | 0.426 | -2.253 | | 0.024* | -1.549 | | 0.132 |
| L4 (%) | -0.634 | 0.557 | -0.352 | | 0.756 | -0.563 | | 0.605 |
| RF (diff) | -0.599 | 0.557 | -0.211 | | 0.863 | -0.458 | | 0.654 |
| RF (%) | -0.352 | 0.756 | -0.493 | | 0.654 | -0.986 | | 0.349 |
| BF (diff) | -0.070 | 0.973 | -0.247 | | 0.809 | -0.423 | | 0.705 |
| BF (%) | -1.760 | 0.085 | -0.282 | | 0.809 | -0.493 | | 0.654 |
| TA (diff) | -0.845 | 0.426 | -0.493 | | 0.654 | -0.282 | | 0.809 |
| TA (%) | 0.000 | 1.000 | -1.338 | | 0.197 | -0.352 | | 0.756 |
| MG (diff) | -0.915 | 0.387 | -3.028 | | 0.002** | -2.042 | | 0.043* |
| MG (%) | -0.352 | 0.756 | -2.324 | | 0.020* | -1.549 | | 0.132 |

Abbreviations: FAM, Fugl-Meyer Assessment; JP, joint pain; MF, motor function; MD, middle deltoid muscle; BB, biceps brachii muscle; L2 and L4, erector spinae muscle at L2 and L4 levels; RF, rectus femoris muscle; BF, biceps femoris muscle; TA, tibialis anterior muscle; MG, medial gastrocnemius muscle; A, affected side; UA, unaffected side; diff, absolute values of (affected - unaffected); %, ratio of affected/unaffected; z and p, z and p values from the Mann-Whitney U tests.

NOTE: * representing a statistically significant difference with p < 0.05; ** representing a statistically significant difference with p < 0.01.
